# Efficacy of Cognitive Training in Older Adults with and without Subjective Cognitive Decline Is Associated with Inhibition Efficiency and Working Memory Span, Not with Cognitive Reserve

**DOI:** 10.3389/fnagi.2018.00023

**Published:** 2018-02-02

**Authors:** Ramón López-Higes, María T. Martín-Aragoneses, Susana Rubio-Valdehita, María L. Delgado-Losada, Pedro Montejo, Mercedes Montenegro, José M. Prados, Jaisalmer de Frutos-Lucas, David López-Sanz

**Affiliations:** ^1^Department of Cognitive Processes, Complutense University of Madrid, Madrid, Spain; ^2^Department of Methods of Research and Diagnostic in Education, Universidad Nacional de Educación a Distancia, Madrid, Spain; ^3^Laboratory of Cognitive and Computational Neuroscience, Madrid, Spain; ^4^Department of Differential and Occupational Psychology, Complutense University of Madrid, Madrid, Spain; ^5^Center for the Prevention of Cognitive Impairment, Madrid-Salud, Madrid, Spain

**Keywords:** cognitive reserve, executive functions, working memory, cognitive training, efficacy, cognitive status

## Abstract

The present study explores the role of cognitive reserve, executive functions, and working memory (WM) span, as factors that might explain training outcomes in cognitive status. Eighty-one older adults voluntarily participated in the study, classified either as older adults with subjective cognitive decline or cognitively intact. Each participant underwent a neuropsychological assessment that was conducted both at baseline (entailing cognitive reserve, executive functions, WM span and depressive symptomatology measures, as well as the Mini-Mental State Exam regarding initial cognitive status), and then 6 months later, once each participant had completed the training program (Mini-Mental State Exam at the endpoint). With respect to cognitive status the training program was most beneficial for subjective cognitive decline participants with low efficiency in inhibition at baseline (explaining a 33% of Mini-Mental State Exam total variance), whereas for cognitively intact participants training gains were observed for those who presented lower WM span.

## Introduction

Cognitive training (CT) may contribute to delay or to prevent cognitive decline in older adults (Gates et al., 2011), although this finding remains controversial. A recent systematic review and meta-analysis conducted by Smart et al. (2017) through a standardized search in main databases (CINAHL Complete, Cochrane Central Register of Controlled Trials, MEDLINE, PsycINFO, and PsycARTICLES) includes adults aged 55 or more with subjective cognitive decline (SCD) defined using published criteria, who receive non-pharmacological intervention or any control condition, with cognitive, behavioral, or psychological outcomes in controlled trails. They reported a small effect size of non-pharmacologic intervention in older adults with SCD, which was greater than obtained by other intervention types; results also revealed that CT had benefits on objectively measured cognitive functioning in this group.

Cognitive training is based on the idea that the brain function is modifiable even in old age. Factors such as education, occupation attainment, expertise, lifestyle, or fitness have been found to influence the trajectory of cognition throughout life (Kramer et al., 2004). These factors are associated with the concept of cognitive reserve; that is, the ability to optimize an individual’s performance in different tasks through the use of alternative neural circuits (Stern, 2012). Barulli and Stern (2013) suggested that cognitive reserve might be related to a more efficient and flexible brain networks recruitment. In recent years, there has been a considerable interest in understanding the relationship between cognitive reserve and cognition in late adulthood. Many studies have provided evidence that changes in cognition and the underlying brain function that take place in the aging process, are susceptible to modification and/or compensation (Robertson, 2013). Furthermore, other studies have pointed out the relevance of cognitive reserve as a factor which might modulate the efficacy of CT (Robertson, 2013; Mondini et al., 2016).

Besides, the initial level of functioning at baseline (Ranganath et al., 2011) should be considered as it can modulate CT outcomes. In a recent study conducted by Borella et al. (2017) the authors observed a compensation effect, that is, participants with lower baseline vocabulary scores, at an older age, and a weaker working memory (WM) performance benefited more from the training program. However, the role of WM performance varied depending on the transfer tasks considered (a visuo-spatial WM task, a short-term memory tasks, a measure of fluid intelligence, a measure of processing speed and two inhibitory measures). It is also well-known that changes in attention and executive functions related to the aging process have consequences such as information processing slowing, difficulties distinguishing relevant and irrelevant information, or in the simultaneous processing of different types of information (Stine-Morrow et al., 2006). Thus, it is expected that executive functioning at the baseline also modulates cognitive training outcomes. In this sense, some authors have argued that high-functioning individuals might show greater training gains than lower-functioning individuals as a result of their higher level of plasticity (see for example, Bissig and Lustig, 2007). Contrarily, an alternative hypothesis is that lower-functioning individuals will have greater training benefits than high-functioning individuals, because they have a higher learning potential (Olazarán et al., 2004).

In Spain a well-known CT program, called *UMAM* by its name in Spanish (programa de la Unidad de Memoria del Ayuntamiento de Madrid; English translation: Madrid City Council Memory Unit Program; Montejo et al., 2013) has shown benefits in memory in older adults without cognitive impairment, both in the post-training assessment and at 6-month follow-up (Montejo, 2015). However, the effects of this CT program on other cognitive domains have not been yet studied. The present study explores the role of cognitive reserve, executive functions (interference efficiency and cognitive flexibility) and individual WM capacity as factors modulating the efficacy of UMAM program in global cognitive status. Considering that people with SCD might be at an increased risk of developing dementia (Jessen et al., 2014; Mitchell et al., 2014), it is of most interest to investigate the benefits of a CT in this population. As main objective we investigate the role of cognitive reserve, executive functions (cognitive flexibility and inhibition efficacy), and WM span as factors modulating CT outcomes on global cognitive status in both groups. In line with previous studies (Olazarán et al., 2004; Mondini et al., 2016) we expect that cognitive reserve will have a greater relative weight than the other two factors as a predictor of CT outcomes on global cognitive status. Finally, we hypothesized that CT will have better outcomes in participants with lower cognitive reserve at the baseline, especially in SCD older adults.

## Materials and Methods

### Participants

**FIGURE 1:**
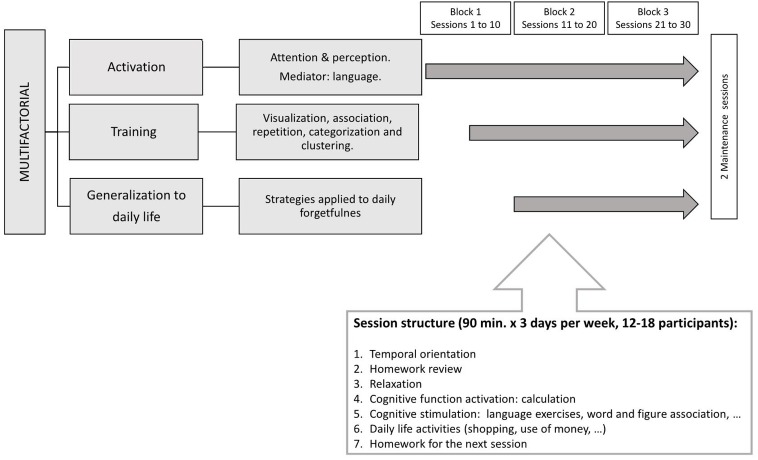
Diagram describing the structure of a session in UMAM program, duration and number of training and maintaining sessions.

Eighty-one older Spanish-speaking adults voluntarily participated in the present study. All of them were recruited from the Center for Cognitive Impairment Prevention (CCIP; Public Health Institute, Madrid City Council), where they were enrolled in the UMAM program. Details about this CT training are provided in Appendix. **Figure 1** summarizes the main features of UMAM program.

Exclusion criteria included: (1) Yesavage Geriatric Depression Scale (GDS-15; Sheikh and Yesavage, 1986) higher or equal to 5; (2) Mini-Mental State Examination (MMSE; Folstein et al., 1975; Spanish adaptation by Lobo et al., 1999) lower than 24 at baseline; (3) Low performance on the Logical Memory delayed recall subtest of the Wechsler Memory Scale – Third Edition (Wechsler, 1997; lower than 10 units for people with 16 years of formal education or more; and lower than 6 units for people with 8–15 years of formal education). All participants had normal or corrected hearing and vision.

Forty-nine participants were identified as older adults with SCD (14 males and 35 females). According to Jessen et al.’s (2014) criteria, these participants: (a) presented self-perception of cognitive decline, mainly associated with memory loss; (b) had requested medical consultation regarding their memory complaints; (c) felt that their subjective decline affected their daily activities; (d) set the onset of their subjective decline within the last 5 years, and (e) concerns associated with their subjective decline were confirmed by a reliable informant. The remaining 32 participants (12 males and 20 females) did not meet criteria for SCD, and they formed a group of cognitively intact older adults (CI).

**Table 1** shows the descriptive statistics for socio-demographic variables, depressive symptomatology, cognitive reserve, executive functions and WM span in both groups at baseline. SCD older adults as a group were less efficient in inhibition (interference) and also presented higher scores in GDS-15 than CI participants. However, it should be taken into account that all subjects had normal scores in this scale. Both groups were equivalent in MMSE scores at the baseline (cognitively intact participants: 28.94 ± 1.19; SCD older adults: 28.35 ± 1.63).

**Table 1:** Descriptive statistics of socio-demographic variables (Age, Years of formal education), cognitive reserve, depressive symptomatology, cognitive flexibility, inhibition efficiency, and working memory capacity (digit reordering) by group.

	Group				*F*(1,79)	*P*
			
	CI		SCD			
				
	Mean	*SD*	Mean	*SD*		
Age	70.94	4.16	71.41	4.83	0.20	0.652
Years of formal education	14.38	5.88	13.13	5.96	0.84	0.362
Cognitive reserve	15.40	3.86	13.50	4.21	3.59	0.063
Yessavage’s Scale	1.53	2.20	3.06	3.09	5.86	0.018
Ratio TMT	2.29	1.08	2.42	0.93	0.30	0.583
Stroop’s Interference	9.06	7.17	4.13	6.63	9.77	0.003
Digit reordering	12.68	2.07	11.87	2.23	2.68	0.105


*In the right side of the table it is shown F-statistic and significance of group differences (Statistical significance criterion: p < 0.05).*

*CI, cognitively intact older adults; SCD, subjective cognitive decline older adults.*

The present study complied with the ethical standards of the Declaration of Helsinki and was approved by the local Ethics Committees of the Participant Institutions.

### Materials

Cognitive reserve was estimated using the Cognitive Reserve Questionnaire (*Cuestionario de Reserva Cognitiva;* Rami et al., 2011), a brief questionnaire suited for clinical context.

The Stroop test (Golden, 1978) and the Trail Making Test parts A and B (TMT-A and TMT-B; Reitan, 1994) were used to assess executive function processes, such as inhibition efficiency and flexibility, respectively. Stroop test provides a measure representing the ability to inhibit an automatized/habitual response (read the word) in favor of an alternative one (color naming). Poor inhibition efficiency has been considered an early sign of cognitive deterioration in the progression to Alzheimer disease. TMT is a common measure of cognitive flexibility and planning ability. Especially, part B is considered a sensitive measurement of cognitive flexibility in geriatric assessment, although it requires other cognitive abilities, such as psychomotor speed and visual scanning. B/A ratio score provides an indicator of executive control according to its correlation with task-switching ability. The assessment protocol also included a digit reordering task (MacDonald et al., 2001), which involves maintaining and manipulating information in WM in contrast with other measures which are only focused on maintenance.

### Procedure

An extensive neuropsychological assessment of each participant was conducted at two different times, one immediately after recruitment (baseline), and then 6 months later (endpoint). Neuropsychological assessments were conducted by an experienced psychologist or psychiatrist at the Center for the Prevention of Cognitive Impairment of Madrid-Salud, a public institution depending on Madrid City Council. In the first session the participant completed the screening tests (MMSE, GDS-15), and the cognitive reserve questionnaire. At the beginning of this first session participants were informed about the main goals of the study and signed an informed consent document. All the remaining neuropsychological tests were applied in a different session. The order in which the tests were presented was randomized in each session for each participant. Neuropsychological tests were applied and scored following the standard instructions provided in the users’ manuals.

### Statistical Analysis

Previous analyses were conducted in order to obtain (1) descriptive statistics by group across measures (pre and post) and across five categories of outcomes (percentages), (2) pre-training differences between groups in MMSE (univariate ANOVA), (3) the effect of UMAM program in the total sample (comparing MMSE scores between pre and post by means of a *t*-test for related measures), (4) a mixed ANOVA (2 groups × 2 measures) with predictors as covariates, and (5) a repeated measures ANOVA for each group in order to explore intragroup differences between pre and post-training measures. Effect size was estimated in the last analysis by means of partial eta-square ($\eta_{p}^{2}$).

Fourteen models were developed for each group by predictor combination, following a procedure described in Mondini et al. (2016). The dependent measure employed in the analyses resulted from subtracting the baseline scores to the endpoint scores in MMSE (post-training).

Regarding predictor variables, we obtained a total score for each participant reflecting an estimation of her/his cognitive reserve. With respect to digit reordering, we considered the number of series correctly ordered by each participant as a measure of his/her WM span. In order to obtain a measure of cognitive flexibility we computed the ratio score part B/part A using data from participants’ TMT, in order to obtain a measure of cognitive flexibility (Lezak, 1995). Finally, regarding the Stroop test, we used the Interference index proposed by Chafetz and Matthews (2004).

All resulting models were compared taking into account a set of indexes providing goodness-of-fit measures: Bayesian Information Criterion (BIC; Schwarz, 1978), Aikake Information Criterion (AIC; Burnham and Anderson, 2002), Bayes factor (BF; Lavine and Schervish, 1999), and *R*^2^. Lower BIC and AIC indexes, as well as higher BF and *R*^2^, are associated with better model fit of the data. IBM SPSS statistical software v. 20.0 was used to obtain BIC, AIC and *R*^2^, but BF was computed with version 0.9.8 of the BayesFactor package developed by Perception and Cognition Lab. Department of Psychological Sciences at the University of Missouri^1^. Once a model has been chosen by means of its goodness-of-fit, we used the statistic *t*-test critical values in order to determine for each variable if it has a significant weight in the prediction or not.

## Results

**Table 2** shows means and standard deviation by group across MMSE measures (pre and post). Once differences in MMSE between post and pre-training were computed for all subjects it was possible to recode values in five categories of training outcomes: (a) a positive value greater or equal than 3 was considered as moderate improvement, (b) positive values equal to 1 and 2 were mild improvement, (c) a value equal to zero meant unchanged performance o null improvement, (d) negative values -1 and -2 formed a category of mild worsening, and (e) negative values greater or equal to -3 points were considered moderate worsening. **Table 2** also summarizes percentages in these five categories by group. There were no statistical differences in percentages across categories between groups, χ^2^(4) = 2.552, *p* = 0.635.

**Table 2:** Descriptive statistics (mean and standard deviation -between parenthesis-) of MMSE by group across measures (pre and post), significance of difference between pre and post in each group, effect size estimation, and percentages of moderate and mild improvement, null improvement, and mild and moderate worsening in MMSE outcomes after training by group.

	MMSE pre	MMSE post	Significance of difference pre–post	Effect size (partial eta-square)
CI	28.97 (1.18)	29.14 (0.83)	n.s	0.030
SCD	28.47 (1.68)	29.22 (1.04)	*p* = 0.025	0.105

	**Outcome in MMSE after training**			
			
CI	% moderate improvement	3.2		
	% mild improvement	32.3		
	% null improvement	38.7		
	% mild worsening	22.6		
	% moderate worsening	3.2		
SCD	% moderate improvement	8.5		
	% mild improvement	36.2		
	% null improvement	36.2		
	% mild worsening	19.1		
	% moderate worsening	0.0		


*CI, cognitively intact older adults; SCD, subjective cognitive decline older adults; MMSE, Mini-Mental State Exam.*

Considering all participants as a group there was a significant improvement from pre to post-training MMSE measure, *t*(80) = -2.46, *p* < 0.02. Groups have very similar MMSE means at the baseline (pre-training measure), *F*(1,79) = 3.119, *p* = 0.81. Results in 2 × 2 mixed ANOVA with covariates pointed out that only the interaction Group × Measure × Stroop’s Interference reached statistical significance, *F*(1,45) = 7.847, *p* < 0.008, $\eta_{p}^{2}$ = 0.148. The interaction Group × Measure × Digit reordering only approached statistical significance, *F*(1,45) = 3.487, *p* = 0.068. There was only a significant difference between MMSE pre- and post-training measures in the SCD group, *F*(1,46) = 5.380, *p* = 0.0025, with a small effect size ($\eta_{p}^{2}$ = 0.105).

### Predictive Models’ Goodness-of-Fit Regarding CT Outcomes in the General Cognitive Status (MMSE)

**FIGURE 2:**
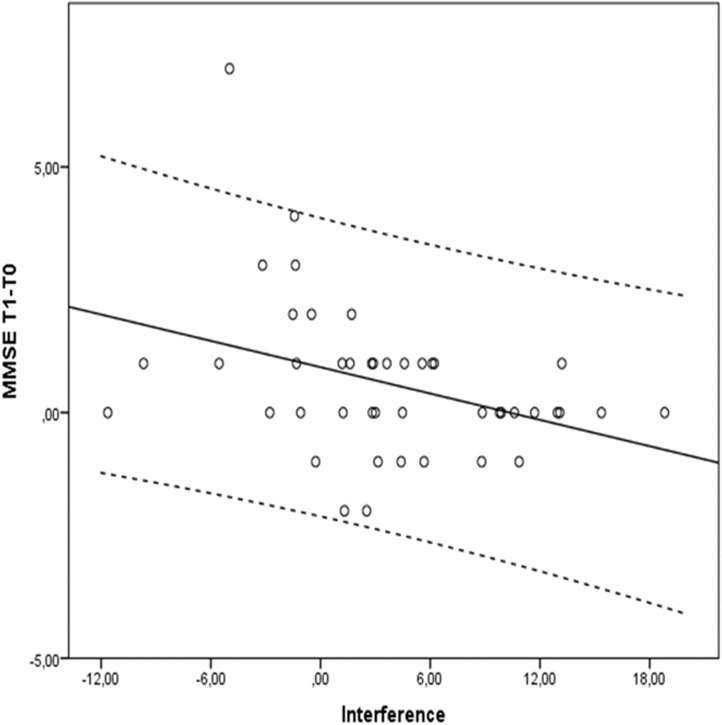
Stroop’s Interference scores and differences between Tl (post-training measure) and T0 (pre-training) measure in MMSE for the SCD group. Dotted lines delimit 95% confidence interval.

**Table 3** shows all possible models involving selected predictors for each group. Models in bold have the best values across goodness-of-fit indexes, that is: lower BIC and AIC, as well as higher BF and *R*^2^, are associated with better model fit of the data. With respect to MMSE, an inspection of **Table 3** underscored that in the cognitively intact group, the best model was the 4th, with digit reordering score as the only predictor (BIC = 102.57; AIC = 98.47; BF = 2.79; *R*^2^ = 0.17; *p* = 0.033). As the results evidenced, the lower the digit reordering score at the baseline was, the greater the benefit of training on cognitive status (MMSE), *t* = -2.41, *p* = 0.023, given that the sign of the statistical test is negative. However, a different picture emerged in the SCD group, since the best model was the 6th, with cognitive reserve and interference as significant predictors (BIC = 119.74; AIC = 113.88; BF = 13.59; *R*^2^ = 0.33; *p* = 0.002). The most influential variable in this case was interference (*t* = -3.74, *p* = 0.001; see **Figure 2**), given that cognitive reserve did not reached statistical significance (*t* = 1.65, *p* = 0.110). As before, the negative sign revealed that the lower Stroop’s Interference score at the baseline was, the better the benefit of training on MMSE.

**Table 3:** Goodness-of-fit of all models predicting UMAM training outcomes in MMSE.

		CI group					SCD group				
			
	Model	BIC	AIC	BF	*R^2^*	*P*	BIC	AIC	BF	*R^2^*	*P*
0	Intercerpt	103.72	100.99				125.42	122.49			
1	CR	107.00	102.90	2.78	0.00	0.760	128.85	124.46	3.00	0.00	0.862
2	TMT	106.90	102.80	2.75	0.00	0.660	128.79	124.39	2.96	0.01	0.760
3	Inter	105.68	101.57	1.10	0.08	0.234	119.15	114.75	3.68	0.13	0.002
4	**DR**	**102.57**	**98.47**	**2.79**	**0.17**	**0.033**	128.50	124.10	3.42	0.00	0.536
5	CR + TMT	110.16	104.69	5.42	0.01	0.860	132.21	126.35	5.91	0.01	0.934
6	**CR + Inter**	109.04	103.57	3.75	0.05	0.492	**119.74**	**113.88**	**13.59**	**0.33**	**0.002**
7	CR+ DR	105.92	100.45	1.33	0.14	0.103	131.97	126.10	5.38	0.02	0.825
8	TMT + Inter	109.00	103.53	2.48	0.09	0.481	122.61	116.74	2.17	0.16	0.008
9	TMT + DR	105.93	100.46	1.13	0.17	0.104	131.86	125.99	7.10	0.01	0.781
10	Inter + DR	105.09	99.63	1.73	0.20	0.068	121.11	115.25	1.50	0.14	0.004
11	CR + TMT + Inter	112.36	105.52	6.93	0.05	0.690	123.16	115.83	5.29	0.33	0.005
12	CR + TMT + DR	109.29	102.45	2.70	0.14	0.209	135.32	127.99	9.29	0.03	0.919
13	TMT + Inter + DR	108.45	101.62	1.17	0.21	0.146	124.56	117.23	1.01	0.17	0.010
14	CR + TMT + Inter + DR	111.71	103.50	3.75	0.17	0.241	125.64	116.84	3.20	0.35	0.009


*CR, cognitive reserve; TMT, Trail Making Test; Inter, Stroop’s interference; DR, digit reordering.*

*BIC, Bayesian Information Criterion; AIC, Aikake Information Criterion; BF, Bayes factor. Models in bold are those which have the best goodness-of-fit in each group.*

## Discussion

UMAM program had a significant benefit on global cognitive status taking all participants as a group. Results based on percentages did not support any association between groups (CI, SCD) and categories of change in cognitive status after training. Given that the clinical significance of mild changes raises doubts, it is more adequate to highlight the difference in the percentage of moderate improvement between CI and SCD groups (3.2 and 8.5% respectively). There were no differences between groups in MMSE pre-training measure. However, further analysis revealed that the interaction between group (CI, SCD) and measure (pre, post) was modulated by Stroop’s Interference, a measure of controlled inhibition efficacy that was considered a relevant predictor of cognitive status outcomes in our study. It is important to pointed out that intragroup differences between pre and post measures revealed that cognitive training was more beneficial for SCD older adults than for CI participants, although effect size was small.

With respect to the main objective of the study, if we consider CT impact in participants’ MMSE (difference between the endpoint and the baseline score) there are different predictor variables that explain the results for each group. In the cognitively intact group a lower WM span at the baseline (score in digit reordering) predicts a positive outcome in global cognitive status (MMSE) after CT. This WM measure explains a 17% of the total variance associated to the measure of change in cognitive status. By contrast, in the SCD group CT outcomes were better for those subjects previously showing low efficiency in inhibition at the baseline. In this case, Stroop’s Interference explains a significant part (33%) of the total variance in MMSE change between the baseline and the endpoint. It must be taken into account that SCD participants showed less ability at the baseline than cognitively intact older adults to inhibit automatized responses (i.e., reading) when trying to name the color. This difference between cognitively intact and SCD older adults in executive performance has been also reported in some previous studies. For example, Kirova et al. (2015) concluded that very early stages of AD are associated with a deficit in executive functions (difficulties with tasks involving divided attention and inhibition of interfering stimuli). Seo et al. (2016) concluded that poor executive functioning (inhibition and goal-directed behaviors) could represent one of the first cognitive signs in the course of AD. They found that a pre-MCI group showed significantly lower scores for visual immediate recall, fluency tests, and Stroop color naming in the Color–Word incongruent condition than the control group. The pattern of results that we observed in SCD older adults seems to show that with respect to a global measure of cognition (the MMSE) the UMAM training program produces larger benefits for individuals with lower efficiency in inhibition at the baseline, and that the variability in dependent variable is explained in a great portion by inhibition efficiency.

Thus, the results obtained contradict our initial predictions, but they are in line with the alternative approach considering that a relevant factor modulating CT benefits is the participant’s level of functioning at the baseline (Ranganath et al., 2011). Accordingly, lower-functioning individuals would benefit more from CT because they have more room for improvement. Our results are also in tune with a recent study conducted by Bamidis et al. (2015) in a community-dwelling sample of cognitively healthy and impaired older adults. The authors used global cognition as a primary measure. This composite score was derived from three different cognitive scores (episodic memory, WM, and executive function), by averaging the *z*-standardized scores. In summary, Bamidis colleagues study showed a robust modulation effect of executive functions’ baseline performance on training (combined physical and cognitive) benefits, that is: the lower the baseline performance, the more benefits were found in global cognition. However, our results differ from others obtained in previous studies providing the relevance of cognitive reserve as a factor that modulates the outcome of a cognitive training program on participant’s cognitive status [Franzmeier et al., 2016 with a sample of amnestic mild cognitive impairment (MCI) patients; Mondini et al., 2016 with a sample of mild to moderate patients with dementia; Olazarán et al., 2004 with MCI and mild to moderate AD patients]. It must be highlighted that SCD older adults had lower cognitive reserve than their cognitively intact peers at the baseline, although such differences only approached statistical significance. This contrast with the results obtained by Mondini colleagues showing that cognitive training has more positive outcome in lower cognitive reserve patients than in higher cognitive reserve patients. Our results also contrast with previous ones showing that educational attainment (proxy of cognitive reserve) modulates the effectivity of training in cognitively normal older adults, being participants with low educational level the group in which outcomes after training are better (Clark et al., 2016).

In line with Lövdén et al. (2010) proposal regarding training efficacy of a mismatch between what they termed the “supply” (one’s capacity for plasticity) and “demand” (the demands required by the environment and one’s capacity for flexibility), a suggestive interpretation of our results might be raised. In this sense, individuals with lower executive functioning and WM span at the baseline have shown larger gains from UMAM training than those with higher level or capacity, perhaps because there was more of a mismatch between the demands of CT and their own capacity for plasticity (supply). Participants with higher previous executive functioning and WM capacity were performing at an optimal level prior to training that is why they may not have profited as much from the type of training employed in our study.

From a practical point of view, these results are remarkably useful since they indicate that a multifactorial CT program (UMAM) is more effective in the SCD group. It might also contribute to the development of alternative programs for others who possibly need different intervention strategies. A follow-up study is currently underway to investigate if the effects of UMAM training are maintained at 12 months.

Finally, as potential limitations of this study, it should be noted that in both groups percentage of females are greater than the corresponding of males, and also sample size could be extended in future studies to strengthen conclusions. Additionally, we have considered small changes as improvement or worsening, therefore we must be cautious when interpreting the results, since these changes could be caused by measurement errors, regression to the mean or practice effects. A relevant future direction would be to explore if the pattern of results obtained could change with long-term interventions.

## Ethics Statement

This study was carried out in accordance with the recommendations of Ethical Commitee of the San Carlos Clinical Hospital (Madrid), which is one of the main Medical Institution that participates in the research project (PSI2015-68793-C3-1,2,3-R), with written informed consent from all subjects. All subjects gave written informed consent in accordance with the Declaration of Helsinki. The protocol was approved by the Ethical Commitee of the San Carlos Clinical Hospital.

## Author Contributions

RL-H, MTM-A, and SR-V: study concept and design, analysis, and interpretation. MLD-L, PM, and MM: preparation of paper and critical review. JMP, JdF-L, and DL-S: interpretation, critical review, and data coding. All authors contributed in writing the manuscript.

## Conflict of Interest Statement

The authors declare that the research was conducted in the absence of any commercial or financial relationships that could be construed as a potential conflict of interest.

## Footnotes

1 http://pcl.missouri.edu/bf-reg
