# Supplementary material for: Efficacy of Cognitive Training in Older Adults with and without Subjective Cognitive Decline Is Associated with Inhibition Efficiency and Working Memory Span, Not with Cognitive Reserve
Source: Front Aging Neurosci. 2018 Feb 2;10:23. doi: 10.3389/fnagi.2018.00023 (PMC5801297; doi:10.3389/fnagi.2018.00023)
Supplement: Supplementary file 1 [file Data_Sheet_1.docx]

**APPENDIX**

**UMAM cognitive training program**

UMAM program is totally free for older adults over 65 in the city of Madrid, but there is a waiting list (no longer than four months) due to great demand it has. Individuals have the possibility to re-participate in the training program one year after finishing their first training period, and provided they have not evolved into some form of dementia. The objective of the UMAM program is to teach basic concepts about memory and promote a higher sense of memory control. The training intends to stimulate cognitive processes and to develop strategies to manage daily-life memory problems (Montejo, Montenegro, Reinoso, De Andrés, & Claver, 2006). Training takes place in groups of 12-18 participants and is supervised by one instructor. It includes 30 sessions, 90 minutes each. There are also two additional maintaining sessions within the next six months.

The first part of each session starts with a review of previous homework, 15 minutes of relaxation and 10 minutes dedicated to randomly ask participants to answer aloud arithmetic problems. The second part, which lasts 60 minutes, involves structured training with activity-sheets, role-playing, audio-visual material, and explanations by an instructor. The outline of each exercise includes motivation, presentation of the specific objectives, individual or group solution, thinking over the results and application to daily life. The last 5 minutes are used to suggest homework. The program is divided into four different modules:

*1) Cognitive Stimulation and Learning of Specific Strategies.* Cognitive processes such as attention, perception or language are stimulated by a set of exercises. Most important, memory strategies are also trained in order to remember places, texts, images and conversations. These strategies include visualization, association, categorization or information elaboration.

*(2) Memory Concepts.* Main aspects of memory functioning, especially those that may have a greater impact on daily life, are presented to participants: memory categories, processes, memory in the elderly and factors impairing memory performance.

*(3) Management of Everyday Memory Failures.* Daily forgetfulness and memory complaints that disturb the participant daily life are extensively treated through role-playing and specific exercises, focusing on prospective memory, retrospective memory, automatic actions (i.e.: taking medication), name retrieval, and memory of texts. The use of external aids (agenda, alarm, telephone book, etc.) is also exercised.

*(4) Meta-memory training.* The program stimulates awareness of memory failures and the mechanisms that each person uses. Negative stereotypes about memory in the elderly are addressed through dialogue and continuous messages are sent out on favorable outcomes when appropriate strategies are used.
